# Editorial: Reviews in hematologic malignancies

**DOI:** 10.3389/fonc.2022.1105523

**Published:** 2022-12-22

**Authors:** Francesco Saraceni, Sonia Morè

**Affiliations:** Hematology Department, Azienda Ospedaliero Universitaria Ospedali Riuniti, Ancona, Italy

**Keywords:** hematologic malignancies, diffuse large B cell lymphoma, reviews, metabolic dysregulation, multiple myeloma

Hematological malignancies are a group of heterogeneous diseases that have always formed the basis of a very active research field in terms of diagnostic approaches, prognostic stratification models, and therapeutic weapons, aiming to achieve personalization of care to the individual patient. Major steps forward have been taken in terms of the study of tumor cells, but increasing attention has been paid in recent years to the tumor microenvironment.

In this collection, titled “Reviews in hematologic malignancies,” three out of the nine reviews pertain to biological insights into hematological malignancies, treating such current and important themes as tumor-associated macrophages (TAMs) and alterations in calcium homeostasis, and highlighting the importance of a biological–metabolic characterization of hemopathies. In particular, Xie et al. describe the novel role of TAMs in the proliferation of malignancies, not only oncological but also hematological. The authors differentiate two phenotypes: M2 TAMs have a low antigen-presenting capability and are involved in angiogenesis, tumor cell invasion, resistance to therapy, and release of anti-inflammatory molecules; in contrast, M1 TAMs may provoke a Th-1 response and secrete pro-inflammatory molecules. The authors describe the clinical–biological implications of their findings in relation to several hemopathies, also proposing potential future therapeutic targets. Immanuel et al. focus on metabolomics, metabolic dysregulation having also emerged in recent years as a hallmark of several hematologic malignancies and a contributor to tumor initiation, progression, metastasis, and drug resistance. The authors demonstrate, using data collected from databases on lymphoid and myeloid malignancies, that calcium could be considered to be a second messenger controlling a wide range of cellular functions, highlighting for the first time the importance of alterations to calcium homeostasis in hematological malignancies and their genetic basis, and thus paving the way for future avenues of research into new metabolic therapeutic pathways.

Moreover, the reviews presented in this Research Topic address lymphoproliferative and myeloproliferative diseases separately, covering current hot topics in each case. Firstly, Mendeville et al. propose a genomic classification of diffuse large B cell lymphomas (DLBCL), outlining a plan of work toward the construction of this genomic classification, harmonization of each classification with others, and translation of this harmonization into clinical practice in order to improve diagnosis and therapy decisions. Recognizing the role of the deregulation of F-box and WD-repeat domain-containing protein 7 (FBW7) as a key component of UPS proteins and a critical tumor suppressor in human cancers, Wan et al. separately examine its role in various hematological malignancies. In particular, they note that FBW7 mutations or inactivation are described as drivers of chemoresistance and poor prognosis, but clinical trials are needed to confirm these data and develop novel therapeutic strategies against them.

With regard to multiple myeloma (MM), there is currently an unmet medical need for improved identification of high-risk cases and the tailoring of effective treatments for these, considering that high-risk patients have been demonstrated to have poorer outcomes than the MM population as a whole. Among the clinical features of high-risk disease, renal failure is one of the most challenging in clinical practice. Xing et al. highlight the available data on the role of HCO hemodialysis, whose role is still controversial. Data from observational or randomized trials demonstrate that its use could reduce free light chains on serum plasma, but may not significantly improve outcomes. There seems to be a trend toward more positive renal outcomes, but data from randomized trials are needed to demonstrate this and to prove the existence of a tangible and significant advantage.

With regard to acute myeloid leukemia (AML), the authors of several reviews tackle specific areas of interest with the objective of improving customization of therapeutic programs for individual patients and widening the applicability of personalized medicine. Wang et al. focus on nucleophosmin (NPM1), which is the most commonly mutated gene in adult AML, presenting either alone or along with other mutations. The authors describe the results of multiple trials employing different therapies in this context, highlighting the future role of association therapy in NPM1-mutated AML and the key role of its use in MRD assessment. The observation that high CD38 expression occurs in AML blasts without an obvious correlation with cytomorphological and genetic characteristics, and that targeting CD38 has demonstrated efficacy and interesting power in modulation of immune surveillance, paves the way for ongoing trials employing anti-CD38 treatments in AML. In particular, Zhong and Ma describe the exploration of novel anti-CD38-based therapies in clinical trials for acute leukemias, especially daratumumab in T-ALL. Visani et al. clearly sum up the therapeutic landscape of optimal treatments for younger AML patients, from intensive chemotherapy to targeted therapy, attesting to major improvements in survival and steps toward a curative strategy in this setting.

Finally, there is also movement toward a genomic classification of myeloproliferative neoplasms (MPNs). Rapid advancements have been made in gene sequencing technology over the last decade. Li et al. tackle 8p11 myeloproliferative syndrome (EMS), which is an aggressive hematological neoplasm occurring with or without eosinophilia and caused by a rearrangement of the FGFR1 gene at 8p11-12. The authors describe in detail the genomic mechanisms and classifications of this rare entity, focusing their attention on the role of TKI in this hematological malignancy. However, data from clinical trials involving targeting of FGFR1 will be needed in the future to develop improved prognostication and ways to therapeutically attack EMS.

In conclusion, many findings have emerged from biological studies of hematological malignancies over recent decades, and these findings are being successfully translated into clinical practice, enabling more precise diagnosis and improved outcomes for various hemopathies.

## Author contributions

FS and SM wrote the manuscript draft and reviewed the final manuscript. All authors contributed to the article and approved the submitted version.

